# Effects of aerobic training on physical activity in people with stroke: protocol for a randomized controlled trial

**DOI:** 10.1186/s13063-018-2823-0

**Published:** 2018-08-17

**Authors:** Larissa Tavares Aguiar, Sylvie Nadeau, Raquel Rodrigues Britto, Luci Fuscaldi Teixeira-Salmela, Júlia Caetano Martins, Christina Danielli Coelho de Morais Faria

**Affiliations:** 1 Department of Physical Therapy, Universidade Federal de Minas Gerais (UFMG), Avenida Antônio Carlos, 6627- Campus Pampulha, Belo Horizonte, Minas Gerais 31270-910 Brazil; 2 École de réadaptation, Université de Montréal (UdeM), Montréal, Canada; 3 Centre de recherche interdisciplinaire en réadaptation (CRIR), Institut universitaire sur la réadaptation en déficience physique de Montréal (IURDPM), CIUSSS Centre-sud-de-l’Ile-de-Montréal, Montréal, Canada

**Keywords:** Stroke, Aerobic exercise, Physical activity, Sedentary lifestyle, Walking, Health

## Abstract

**Background:**

Post-stroke physical inactivity is explained by several factors related to the stroke, which have been suggested as the causes and consequences of functional declines and health problems. Therefore, it is important to increase physical activity levels and reduce the time spent in low-energy expenditure activities after a stroke. Since the maintenance of cardiorespiratory fitness is a significant predictor of physical activity levels post-stroke, it may be important to investigate whether aerobic training is effective in increasing physical activity levels and reducing the time spent in low-energy expenditure activities in this population. The efficacy of aerobic training on these variables is not well known. The primary objective of this trial will be to investigate the effects of aerobic treadmill training on physical activity levels and on time spent in low-energy expenditure activities in people with stroke. The secondary aim will be to evaluate the effects of the training on cardiorespiratory fitness, endurance, depression, mobility, quality of life, and participation.

**Methods/design:**

A randomized controlled trial, with blinded assessments, will be performed in a community-based setting. Altogether, 22 adults with a diagnosis of stroke (>6 months) who are sedentary or insufficiently active will be included. Participants will be randomly assigned to either: (1) aerobic treadmill training (experimental group, at 60–80% of their heart rate reserve) or (2) walking outside (control group, below 40% of heart rate reserve). Both groups will attend 40-min training sessions, three times per week over 12 weeks, in groups of two to four participants, with a trained physiotherapist. Primary outcomes are physical activity levels and time spent in low-energy expenditure activities (Multi-sensor SenseWear Mini® and Human Activity Profile). Secondary outcomes are cardiorespiratory fitness (peak oxygen uptake VO_2peak_ and ventilatory threshold), endurance, depression, mobility, quality of life, and participation. The effects of the training will be analyzed from the collected data using intention to treat. Between-group differences will be measured by two-way repeated measures ANOVA, considering the baseline, post-training, and 4-week follow-up.

**Discussion:**

The results of this trial will likely provide valuable new information on the effects of aerobic treadmill training on physical activity levels and on time spent in low-energy expenditure activities of individuals with stroke, through changes in cardiorespiratory fitness.

**Trial registration:**

ClinicalTrials.gov, NCT02798237. Registered on 13 June 2016.

**Electronic supplementary material:**

The online version of this article (10.1186/s13063-018-2823-0) contains supplementary material, which is available to authorized users.

## Background

Stroke has a high prevalence worldwide [1] and people with stroke are more likely to require help with mobility, self-care, and household activities [2]. Furthermore, they are 40% more likely to have limitations in performing activities compared to matched controls [2]. Furthermore, people with stroke are at high risk of being affected by other cardiovascular diseases or recurrent stroke, which are often associated with the severity of the stroke [1]. Thus, it is important to develop and implement interventions to prevent and manage the associated post-stroke disabilities and the complications and risk factors associated with recurrent stroke, and to promote functionality [3].

Physical activity has the potential to influence several functional domains and health status in individuals with stroke [3]. Physical activity is defined as any bodily movements produced by the skeletal muscles that result in energy expenditure, such as those performed during activities of daily living at home, at work, during leisure, or transport [4]. Exercise is a type of physical activity with specific characteristics: it is repeatedly performed, in a planned and structured way, to improve or maintain physical fitness [4]. Sedentary behavior, which is part of the physical activity continuum and has an independent impact on health, was recently defined as any behavior performed while awake that involves energy expenditure ≤1.5 metabolic equivalents (METs) while in a sitting, reclining, or lying posture [5].

People after stroke have low physical activity levels and spend more time in low-energy expenditure activities, compared with matched individuals without stroke [6]. After a stroke, individuals spend on average 80% of their time in low-energy expenditure activities [7]. Only 15% engage in light and 5% in moderate-to-vigorous intensity physical activity [7]. Post-stroke physical inactivity is explained by several factors, which are directly and indirectly related to the stroke and have been suggested as the causes and consequences of functional declines and health problems [1, 3, 6, 7]. After a stroke, low physical activity levels are the main consequences of the concomitant presence of cardiovascular diseases and disabilities, such as reduced cardiorespiratory fitness (median of 14 mL kg^-1^ min^-1^, ranging from 8 to 23 mL kg^-1^ min^-1^) [8–10], depression [3], mobility limitations [11], as well as low perception of quality of life [12] and restricted social participation [2]. Besides contributing to physically inactive and sedentary lifestyles, these disabilities can also be aggravated by physically inactivity [3], creating a vicious cycle that dramatically impedes post-stroke individuals in adopting healthy lifestyles. There is evidence that both physical inactivity and time spent in low-energy expenditure activities are risk factors for developing diabetes mellitus and cardiovascular diseases (including stroke), and for overall mortality [13, 14]. For every 25–30 min increase in sedentary time per day, there is a 1% increase in risk of cardiovascular disease for elderly individuals [13]. Therefore, it is important to increase physical activity levels and reduce the time spent in low-energy expenditure activities after stroke.

Investigating the influence of exercise on physical activity levels and the importance of reducing the time spent in low-energy expenditure activities in people with stroke has been suggested in a scientific statement from the American Stroke Association as a future research direction [3]. Any exercise would probably increase physical activity level and reduce sedentary duration on the same day that it is performed. However, it is not known which intervention could effectively improve physical activity. In addition, it is also unknown whether these changes occur in individuals with different baseline levels of physical activity and time involved in low-energy expenditure activities, nor if they are maintained after the cessation of the intervention. Since the efficacy of aerobic training in improving cardiorespiratory fitness in people with stroke has been shown [8–10] and that cardiorespiratory fitness is a significant predictor of physical activity after stroke [15], it may be important to investigate whether aerobic training is effective in increasing physical activity levels and reducing the time spent in low-energy expenditure activities in this population. However, the efficacy of aerobic training on these variables is not well known [3, 9].

Only four randomized controlled trials have investigated the effects of aerobic training on physical activity levels in people with stroke [16–20]. In three trials, no between-group differences were found [17, 18, 20]. In the other trial, the physical activity levels of the experimental group (aerobic training plus lower-limb resistance training and home exercises) improved compared to the control group (no intervention) [19]. Therefore, it is not known whether aerobic training alone is an effective intervention. In addition, in all four trials [17–20], physical activity was not the primary outcome and, in three of these trials, physical activity was assessed using self-reported questionnaires [17–19]. Secondary outcomes are not confirmatory, but exploratory; thus, it is not possible to give much credence to them [21]. Although questionnaires are inexpensive and easy to use [22], their accuracy is questionable, since they can be influenced by recall bias, social desirability bias, and the inability of the participants to estimate the frequency, duration, and intensity of their physical activity [3, 22]. One of the four trials assessed the effects of aerobic training on physical activity levels of people with stroke by the number of steps taken over 48 h measured by an accelerometer [20]. However, there is some doubt about whether the number of steps alone is an adequate measure of physical activity levels, since it does not provide any information regarding important parameters of physical activity, such as intensity or duration [15, 23]. Furthermore, the number of steps does not provide information on upper limb or cycling activities [4]. According to a systematic review, although there is no gold-standard portable monitor that can assess physical activity, a multi-sensor device, such as the one that will be used in the present study, has the potential to improve accuracy, since the data are estimated by multiple sensors [22]. In addition, a multi-sensor device can provide information on frequency, duration, and the intensity of physical activity, including low-intensity and non-ambulatory activity [22–26]. To our knowledge, only one randomized controlled trial has investigated the efficacy of an intervention that was not aerobic training in decreasing the time spent in low-energy expenditure activities in individuals with stroke [24]. The experimental group received four counseling sessions with a message of “sit less and move more,” whereas the control group received the same number of counseling sessions but on calcium intake for bone health [24]. However, no statistically significant between-group differences were found [24]. Therefore, measuring physical activity levels and the time spent in low-energy expenditure activities as primary outcomes using adequate measures, as planned for the present study, is recognizably important in trials with people with stroke [3].

Thus, the primary aim of this trial is to investigate the efficacy of aerobic treadmill training in improving physical activity levels and reducing the time spent in low-energy expenditure activities of people with stroke. The secondary aim is to evaluate the effects of the training on cardiorespiratory fitness, endurance, depression, mobility, quality of life, and participation.

## Methods/design

**Fig. 1:**
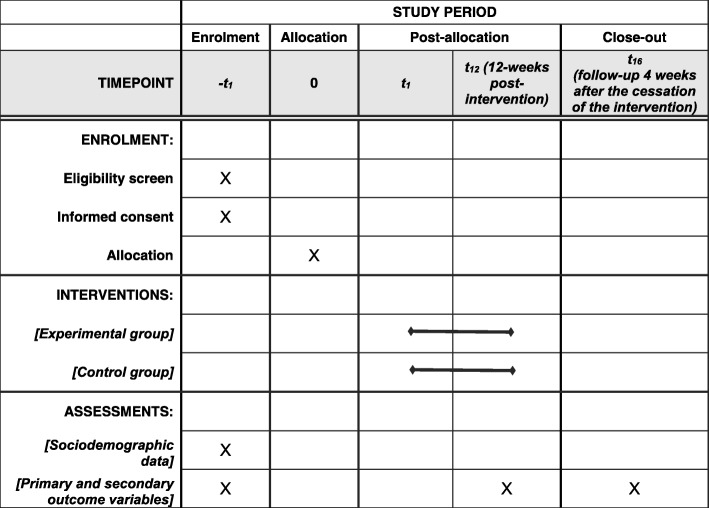
Schedule of enrolment, interventions, and assessments

**Fig. 2:**
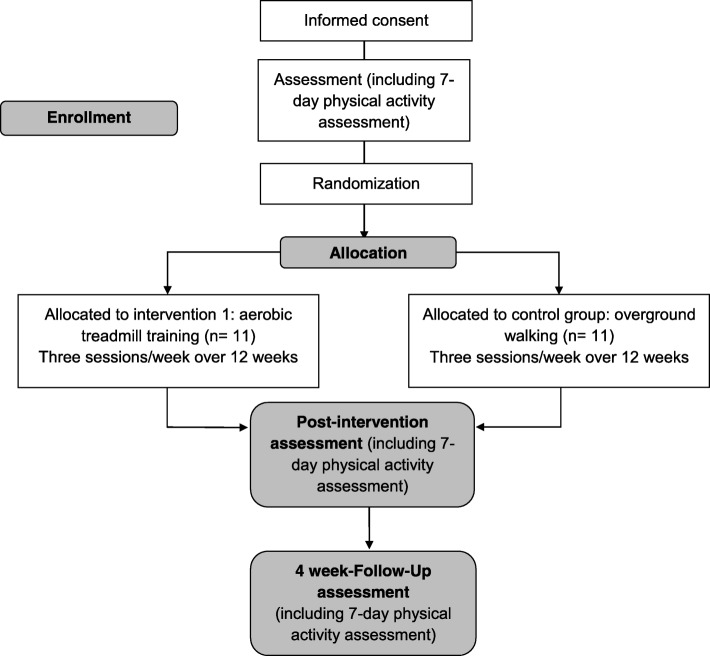
Flow diagram of the study

A superiority parallel-group randomized controlled trial, with concealed allocation and blinded assessments, will be carried out. A trained researcher, blinded to the group allocation, will collect the written consent and the outcome measures at baseline, post-intervention (after the 12-week intervention), and at the 16-week follow-up, i.e., 4 weeks after the cessation of the intervention (Fig. 1). The examiner will be blinded to group allocation, and the participants and the treating physiotherapist will be asked not to share any information about the intervention with the examiner. Moreover, evaluations will be carried out in different places. Participants will be randomly assigned to either (1) aerobic treadmill training (experimental group) or (2) walking outside (control group) (Fig. 2).

This randomized controlled trial was prospectively registered at https://clinicaltrials.gov/ct2/show/NCT02798237 (NCT02798237) and received approval (51454115.6.0000.5149) from the institutional ethical review board. The study commenced in August 2017 and the estimated completion date is December 2018.

### Setting

This study will be carried out in a community-based setting in Belo Horizonte, Brazil.

### Participants

Individuals will be recruited from the general community, by contacting health centers and research groups. They will be included if they are ≥20 years of age, have a diagnosis of stroke (>6 months), are inactive or insufficiently active [27], and have written medical permission to allow them to participate in the study. The classification of the Centers for Disease Control and Prevention will be used to determine if an individual is inactive or insufficiently active [27]. Participants will be asked about the exercises they performed most often over the last 4 weeks, including their frequency and duration [28]. Individuals who report not having practiced any exercise over the last month will be classified as inactive [27]. Those who report that they have performed physical exercise over the last month for at least five times per week for more than 30 min at a moderate intensity or for at least three times per week for at least 20 min at a vigorous intensity will be classified as having moderate or vigorous exercise levels, respectively [27]. The intensity of the exercises reported by the participants will be determined based upon the estimated MET [27]. Exercises performed at vigorous intensity are those with an assigned MET that is greater than 60% of the maximum cardiorespiratory capacity of the individual [27]. To determine 60% of the maximum cardiorespiratory capacity, we use 0.6 × (60 – 0.55 × age) / 3.5 for men and 0.6 × (48 – 0.37 × age) / 3.5 for women [27]. Individuals classified as having moderate or vigorous exercise levels will be excluded from the study. Individuals who report doing physical exercise over the last month that is not classified as vigorous or moderate intensity will be classified as insufficiently active [27]. Exclusion criteria are: (1) cognitive impairments, as determined by the education-adjusted cut-off scores for the Mini-Mental Status Examination depending on the education level of each participant (illiterate: 13 points; elementary and middle school: 18 points; and high-school: 26 points) [28]; (2) an inability to respond to simple verbal commands [29]; (3) an inability to walk independently for at least 10 min, with or without a walking device; and (4) have pain or other disorders precluding their participation.

### Participant withdrawal

Participants may withdraw from the trial for any reason at any time. The investigator can withdraw participants from the study for safety purposes. Missing a maximum of six consecutive sessions will be allowed.

### Randomization procedures

The randomization sequence will be computer generated prior to the commencement of the study by a trained research assistant, who will be not involved in the study, and maintained in randomized blocks in sequentially numbered sealed opaque envelopes. Eligible participants will be randomly allocated to either the experimental or control group, after the baseline measurements. The training therapist will be responsible for revealing the contents of the sealed opaque envelopes, and therefore, for revealing the allocation.

### Intervention and control

All participants will attend three 40-min sessions per week over 12 weeks [3, 9, 30], in groups of two to four. A trained physiotherapist who has experience with aerobic training will supervise both groups. The exercise intensity will be determined, based upon the results of the cardiopulmonary exercise test [3, 9].

Before and after training, the participants will remain at rest for 10–15 min, during which time their heart rate (heart rate monitor), blood pressure (aneroid sphygmomanometer and stethoscope), and peripheral oxygen saturation (oximeter) will be measured. Heart rate will be continuously monitored. Participants will be asked to report any discomfort and not to undertake any other exercise program during their participation in the present study.

### Experimental group

The participants in the experimental group will perform 5 minutes of warm-up and cool-down treadmill walking, followed by 30 min of aerobic treadmill training at 60–80% of their heart rate reserve [3, 9]. However, for those with poor exercise tolerance, short exercise bouts (for a minimum of 10 min, until they have completed 30 min) may be given initially, with interspersed rest periods [3, 9]. As their exercise tolerance improves, longer periods of continuous exercise with shorter rest periods will be implemented [3, 9]. The progression of the intensity of the treadmill training will be individualized, depending upon the individual’s ability, rate of perceived exertion, heart rate, and blood pressure responses [9]. If they are exercising on the treadmill below their cardiac training range, they will be requested to increase their speed until they are in their cardiac training zone.

### Control group

The participants in the control group will walk outside at a comfortable pace at <40% of their heart rate reserve. If they are exercising above 40% of their heart rate reserve, they will be requested to slow down, until their training zone reaches ≤40%. If the efficacy of the intervention is proved, the experimental intervention will be offered to the participants of the control group at the end of the study.

### Procedures

A trained researcher, who will be blinded to the group allocation, will collect the sociodemographic data and all outcomes.

### Primary outcome measures

The primary outcomes will include both physical activity level and time spent in low-energy expenditure activities, as they are part of the physical activity continuum. Both will be measured by an objective device, the multi-sensor SenseWear Mini®. In addition, physical activity level will be also assessed by a subjective method, the Human Activity Profile (HAP), since objective and subjective methods are complementary, and the questionnaire has the advantage of higher clinical applicability [23, 29].

The multi-sensor SenseWear Mini® (BodyMedia, Pittsburgh, PA, USA; software version 8.1) provides objective measurements of physical activity levels and time spent in low-energy expenditure activities [23]. It is a portable, non-invasive, and lightweight activity monitor [23]. The validity of this multi-sensor has already been established for the measurement of physical activity, compared with indirect calorimetry and double-labeled water [26, 31, 32]. The data acquired by the multiple sensors (heat flux, skin temperature, galvanic skin response, and triaxial accelerometer) are integrated with clinical characteristics (age, height, body mass, sex, and smoking habits) into an algorithm that estimates physical activity levels and time spent in low-energy expenditure activities [23]. This monitor can measure the intensity, frequency, and duration of physical activity and is able to detect improvements in physical activity levels in longitudinal studies with people with stroke [23]. Average daily energy expenditure, expressed in kilocalories, will be used to estimate physical activity levels. The average time spent each day being sedentary, expressed as a percentage of total waking time, will be used to estimate the time spent in low-energy expenditure activities (≤1.5 METs). The participants will use this device, attached to the back of a non-paretic arm [23], for 7 days during each assessment period to reduce the possibility of bias related to differences in physical activity levels and time spent in low-energy expenditure activities for each day during the period [22]. There will be three assessment periods: baseline, post-intervention (after the 12-week intervention), and the 16-week follow-up.

The HAP, which provides subjective measures of physical activity levels [29], will be administered by interviews [29]. It asks about 94 activities, which are hierarchically graded according to their metabolic equivalent [29]. These activities include personal care, transportation, home maintenance, social and leisure activities, and exercise [29]. The HAP Adjusted Activity Score is calculated by subtracting the number of activities that the participant has stopped doing from the number of the last item on the list that they still doing [29]. The Adjusted Activity Score is a better estimate of average energy expenditure spent by an individual [29]. The HAP is commonly used in studies with people with stroke and has been shown to be suitable for assessing physical activity levels in this population [29].

### Secondary outcome measures

Secondary outcomes will be cardiorespiratory fitness (cardiopulmonary exercise test); endurance (six-minute walk test or 6MWT and shuttle-walk test or SWT); depression (Patient Health Questionnaires PHQ-2 and PHQ-9); mobility (comfortable and maximum gait speeds); quality of life (Stroke-Specific Quality of Life Scale or SSQOL); and participation (Stroke Impact Scale or SIS). All measures are suitable for evaluating post-stroke individuals [33–40].

Cardiorespiratory fitness (peak oxygen uptake VO_2peak_ and ventilatory threshold) will be measured using a cardiopulmonary exercise test with a gas analyzer (CPX Ultima Medical Graphics®, USA) and an electrocardiogram. This is an objective, non-invasive, and widely used test, and it is considered to be the gold standard for evaluating cardiorespiratory fitness [35]. The cardiopulmonary exercise test is feasible and safe for people with stroke [3, 41]. The test will use an electronic treadmill, with a progressive ramp protocol [42]. It will follow the recommendations of the American College of Sports Medicine [35]. All tests will be monitored by a cardiologist, who has advanced life support training.

Endurance will be measured using the 6MWT and the SWT [38, 39]. Although the cardiopulmonary exercise test is the gold standard for measuring aerobic capacity, its use in clinical practice is limited, due to the need for specialized equipment and trained personnel [35, 38]. Clinical alternatives are sub-maximal exercise tests, such as the 6MWT and the SWT. These tests are simple and inexpensive, and do not require advanced training [38, 39]. For the 6MWT, the maximum distance covered will be measured [39]. The SWT is an incremental test that consists of 12 levels whose speed is set by an audible signal. It is held in a 10-m track [39].

Depression will be assessed by the PHQ-9 and PHQ-2 [36]. The PHQ-9 is used to assess the frequency of nine depressive symptoms over the previous 2 weeks [36, 43]. The PHQ-2 includes only two of the nine questions [36, 43]. As recommended, the PHQ-9 will be applied, by interview, only for those participants who have a positive outcome on the PHQ-2 [36, 43].

Mobility will be evaluated by both comfortable and maximum gait speeds during a 10-m walk test [38]. The instructions will be standardized and only one trial will be employed [44, 45].

Quality of life will be measured using the SSQOL [34], which consists of 49 items distributed into 12 domains (energy, family roles, language, mobility, mood, personality, self-care, social roles, thinking, upper extremity function, vision, and work/productivity) [34]. The SSQOL is applied by interviews and does not take long to administer [34].

Participation will be measured by the social participation section of the SIS 3.0 [37]. The items on participation are evaluated in terms of frequency of self-reported participation restriction over the previous month [37].

### Data monitoring committee

The study will not have a data monitoring committee, since aerobic training rarely has adverse effects [9]. However, participants will be monitored during the exercise sessions, to identify any kind of signals or symptoms, such as pain, dizziness, and loss of balance, that would require interruption of the exercise session or their exclusion from the study. In addition, participants will be asked to report any discomfort, which will be registered and reported.

### Sample size calculation

The sample size was based on the only previous randomized controlled trial that we found that measured changes in physical activity levels of people with stroke associated with aerobic exercise training [16]. The effect size was derived from the study of Teixeira-Salmela et al. [19], who assessed changes in physical activity levels with HAP Adjusted Activity Scores that were associated with aerobic plus lower-limb strengthening training and home exercises, for people with chronic stroke. In that study, the experimental group (*n* = 6) showed an average increase in Adjusted Activity Score of 20 ± 6.1 after the intervention, whereas the control group (*n* = 7) had an average reduction of 1.86 ± 0.19 points. To be able to detect a between-group difference of 20 points on the HAP Adjusted Activity Score, considering a significance level of 5% and a desired power of 80%, nine participants per group are required (a total of 18 participants) [46]. Assuming an expected dropout rate of 20%, a target of 22 participants was set (11 participants per group).

### Statistical analyses

Each participant will be assigned a unique ID. Two independent examiners, who will be blinded to the group allocation, will enter the data into a computer and verify any missing or apparently wrong values. The original paper forms will be kept in a secure place. The electronic files will be available only to the research team.

An independent examiner, who will be blinded to group allocation, will perform the statistical analysis, using the software SPSS (SPSS Inc., Chicago, IL, USA). Descriptive statistics will be calculated for all outcomes.

The effects of the interventions will be analyzed from the collected data using intention to treat. Data from the last available assessment will be used for missed sessions. Between-group differences will be evaluated using two-way repeated measures ANOVA, considering the baseline, post-training, and follow-up measurements. If there are baseline differences between the groups, analysis of covariance will be used to eliminate the influence of extraneous factors, such as baseline cardiorespiratory fitness, physical activity levels, and time spent in low-energy expenditure activities. The level of significance will be set at 5% and adjusted for multiple comparisons. Data distribution and equality of variance will also be analyzed, to ensure the parametric analysis has been applied correctly.

## Discussion

Although the efficacy of aerobic training in improving VO_2peak_ in individuals with stroke is well known, it is unknown if this training improves physical activity levels and reduces the time spent in low-energy expenditure activities. In fact, it is still unclear which interventions can improve physical activity levels and reduce time spent post-stroke in low-energy expenditure activities. Therefore, the results of this randomized controlled trial will likely provide valuable new information regarding the effects of aerobic treadmill training on physical activity levels and time spent in low-energy expenditure activities for individuals with stroke. Since low levels of physical activity are associated with risk of cardiovascular disease, the investigated intervention may help to improve functionality and health status and reduce the burden of care on the families of people with stroke. Whether exercise improves function and quality of life and prevents secondary diseases, such as stroke, is a high priority research objective, which may be answered by this study [47].

Considering that previous randomized controlled trials have rarely investigated the effects of aerobic training on physical activity levels and that none have investigated these effects on the time spent in low-energy expenditure activities or have used a multi-sensor monitor to measure these outcomes, this study may increase the use of evidence-based practice in that domain, and, hence, may improve the care of people with stroke.

This trial design does have some limitations. It will include a convenience sample, which may limit generalizability. Furthermore, both the participants and the physiotherapist, who will provide the interventions, will not be blinded (Additional file 1).

### Trial status

Protocol version: 1

Trial registration: ClinicalTrials.gov

Registration number: NCT02798237.

Date of trial registration: 13 June 2016

Was this trial prospectively registered? Yes

Date recruitment began: August 2017. Recruiting is ongoing

Anticipated completion date: December 2018

## Additional file

Additional file 1: SPIRIT Checklist. (DOCX 49 kb)

## Abbreviations

6MWT: Six-minute walk test
HAP: Human Activity Profile
MET: Metabolic equivalent
PHQ: Patient Health Questionnaire
SIS: Stroke Impact Scale
SSQOL: Stroke-Specific Quality of Life Scale
SWT: Shuttle-walk test
VO_2peak_: Peak oxygen uptake

## References

[1] Benjamin EJ, Blaha MJ, Chiuve SE, Cushman M, Das SR, Deo R (2017). Heart disease and stroke Statistics-2017 update: a report from the American Heart Association. Circulation.

[2] Skolarus LE, Burke JF, Brown DL, Freedman VA (2014). Understanding stroke survivorship: expanding the concept of poststroke disability. Stroke.

[3] Billinger SA, Arena R, Bernhardt J, Eng JJ, Franklin BA, Johnson CM (2014). Physical activity and exercise recommendations for stroke survivors: a statement for healthcare professionals from the American Heart Association/American Stroke Association. Stroke.

[4] Caspersen CJPK, Christenson GM (1985). Physical activity, exercise, and physical fitness: definitions and distinctions for health-related research. Public Health Rep.

[5] Tremblay MS, Aubert S, Barnes JD, Saunders TJ, Carson V, Latimer-Cheung AE (2017). Sedentary behavior research network (SBRN) - terminology consensus project process and outcome. Int J Behav Nutr Phys Act.

[6] Butler EN, Evenson KR (2014). Prevalence of physical activity and sedentary behaviour among stroke survivors in the United States. Top Stroke Rehabil.

[7] Joseph C, Conradsson D, Hagstromer M, Lawal I, Rhoda A. Objectively assessed physical activity and associated factors of sedentary behavior among survivors of stroke living in Cape Town, South Africa. Disabil Rehabil. 2017:1–7. 10.1080/09638288.2017.1338761 28625084

[8] Marsden DL, Dunn A, Callister R, Levi CR, Spratt NJ (2013). Characteristics of exercise training interventions to improve cardiorespiratory fitness after stroke: a systematic review with meta-analysis. Neurorehabil Neural Repair.

[9] Pang MY, Charlesworth SA, Lau RW, Chung RC (2013). Using aerobic exercise to improve health outcomes and quality of life in stroke: evidence-based exercise prescription recommendations. Cerebrovasc Dis.

[10] Saunders DH, Sanderson M, Hayes S, Kilrane M, Greig CA, Brazzelli M (2016). Physical fitness training for stroke patients. Cochrane Database Syst Rev.

[11] Faria CD, Teixeira-Salmela LF, Nadeau S (2013). Predicting levels of basic functional mobility, as assessed by the timed “up and go” test, for individuals with stroke: discriminant analyses. Disabil Rehabil.

[12] Polese JC, Pinheiro MB, Machado GC, Faria CDCM, Hirochi TL, Teixeira-Salmela LF (2014). Chronic Hemiparetic subjects with higher physical activity levels report better quality of life. Rev Neuro.

[13] Fitzgerald JD, Johnson L, Hire DG, Ambrosius WT, Anton SD, Dodson JA (2015). Association of objectively measured physical activity with cardiovascular risk in mobility-limited older adults. J Am Heart Assoc.

[14] Young DR, Hivert M-F, Alhassan S, Camhi SM, Ferguson JF, Katzmarzyk PT (2016). Sedentary behavior and cardiovascular morbidity and mortality: A Science Advisory From the American Heart Association. Circulation..

[15] English C, Manns PJ, Tucak C, Bernhardt J (2014). Physical activity and sedentary behaviours in people with stroke living in the community: a systematic review. Phys Ther.

[16] Aguiar L, Nadeau S, Martins J, Teixeira-Salmela L, Britto R, Faria C. Efficacy of interventions aimed to improve physical activity levels in individuals with stroke: a systematic review. Disabil Rehabil. 2018; In press 10.1080/09638288.2018.1511755 30451539

[17] Severinsen K, Jakobsen JK, Pedersen AR, Overgaard K, Andersen H (2014). Effects of resistance training and aerobic training on ambulation in chronic stroke. Am J Phys Med Rehabil.

[18] Shaughnessy M, Michael K, Resnick B (2012). Impact of treadmill exercise on efficacy expectations, physical activity, and stroke recovery. J Neurosci Nurs.

[19] Teixeira-Salmela LF, Olney SJ, Nadeau S, Brouwer B (1999). Muscle strengthening and physical conditioning to reduce impairment and disability in chronic stroke survivors. Arch Phys Med Rehabil.

[20] Ivey FM, Stookey AD, Hafer-Macko CE, Ryan AS, Macko RF (2015). Higher treadmill training intensity to address functional aerobic impairment after stroke. J Stroke Cerebrovasc Dis.

[21] Harvey LA, Glinsky JV, Herbert RD (2018). 50 tips for clinical Trialists. Brain Impair.

[22] Ainsworth B, Cahalin L, Buman M, Ross R (2015). The current state of physical activity assessment tools. Prog Cardiovasc Dis.

[23] Fini NA, Holland AE, Keating J, Simek J, Bernhardt J (2015). How is physical activity monitored in people following stroke?. Disabil Rehabil.

[24] English C, Healy GN, Olds T, Parfitt G, Borkoles E, Coates A (2016). Reducing sitting time after stroke: a phase II safety and feasibility randomized controlled trial. Arch Phys Med Rehabil.

[25] Hiremath SV, Ding D, Farringdon J, Vyas N, Cooper RA (2013). Physical activity classification utilizing SenseWear activity monitor in manual wheelchair users with spinal cord injury. Spinal Cord.

[26] Reece JD, Barry V, Fuller DK, Caputo J (2015). Validation of the SenseWear armband as a measure of sedentary behaviour and light activity. J Phys Act Health.

[27] Centers for Disease Control and Prevention (2001). Physical activity trends - United States, 1990–1998. Morb Mort Week Rep.

[28] Bertolucci PH, Brucki SM, Campacci SR, Juliano Y (1994). The Mini-Mental State Examination in a general population: impact of educational status. Arq Neuropsiquiatr.

[29] Teixeira-Salmela LF, Devaraj R, Olney SJ (2007). Validation of the human activity profile in stroke: a comparison of observed, proxy and self-reported scores. Disabil Rehabil.

[30] Globas C, Becker C, Cerny J, Lam JM, Lindemann U, Forrester LW (2012). Chronic stroke survivors benefit from high-intensity aerobic treadmill exercise: a randomized control trial. Neurorehabil Neural Repair.

[31] Manns PJ, Haennel RG. SenseWear armband and stroke: validity of energy expenditure and step count measurement during walking. Stroke Res Treat. 2012;2012:1–8. 10.1155/2012/247165 PMC3475303 23094200

[32] Moore SA, Hallsworth K, Bluck LJ, Ford GA, Rochester L, Trenell MI (2012). Measuring energy expenditure after stroke: validation of a portable device. Stroke.

[33] American Thoracic Society, American College of Chest Physicians (2003). ATS/ACCP statement on cardiopulmonary exercise testing. Am J Respir Crit Care Med.

[34] Faria CD, Silva SM, Correa JC, Laurentino GE, Teixeira-Salmela LF (2012). Identification of ICF participation categories in quality-of-life instruments utilized in cerebrovascular accident victims. Rev Panam Salud Publica.

[35] Pescatello LS, Arena R, Riebe D, Kluwer P. ACSM’s guidelines for exercise testing and prescription. 9th ed. Philadelphia: Wolters Kluwer; 2014.

[36] Prisnie JC, Fiest KM, Coutts SB, Patten SB, Atta CA, Blaikie L (2016). Validating screening tools for depression in stroke and transient ischemic attack patients. Int J Psychiatry Med.

[37] Tse T, Douglas J, Lentin P, Carey L (2013). Measuring participation after stroke: a review of frequently used tools. Arch Phys Med Rehabil.

[38] Tyson S, Connell L (2009). The psychometric properties and clinical utility of measures of walking and mobility in neurological conditions: a systematic review. Clin Rehabil.

[39] van Bloemendaal M, Kokkeler AM, van de Port IG (2012). The shuttle walk test: a new approach to functional walking capacity measurements for patients after stroke?. Arch Phys Med Rehabil.

[40] Vanroy C, Vanlandewijck Y, Cras P, Feys H, Truijen S, Michielsen M (2014). Is a coded physical activity diary valid for assessing physical activity level and energy expenditure in stroke patients?. PLoS One.

[41] Marzolini S, Oh P, McIlroy W, Brooks D (2012). The feasibility of cardiopulmonary exercise testing for prescribing exercise to people after stroke. Stroke.

[42] Pereira DAG, Samora GAR, Alencar MCN, Vieira DSR, Parreira VF, Pereira LSM (2012). Cardiopulmonary exercise test with ramp protocol in adults with heart failure. Rev Bras Med Esporte.

[43] de Man-van Ginkel JM, Hafsteinsdottir T, Lindeman E, Burger H, Grobbee D, Schuurmans M (2012). An efficient way to detect poststroke depression by subsequent administration of a 9-item and a 2-item patient health questionnaire. Stroke.

[44] Faria CD, Teixeira-Salmela LF, Neto MG, Rodrigues-de-Paula F (2012). Performance-based tests in subjects with stroke: outcome scores, reliability and measurement errors. Clin Rehabil.

[45] Nascimento LRCL, Freitas DC, Morais TM, Polese JC, Teixeira-Salmela LF (2012). Different instructions during the ten-meter walking test determined significant increases in maximum gait speed in individuals with chronic hemiparesis. Braz J Phys Ther.

[46] Cohen J (1988). Statistical power analysis for the Behavioural sciences.

[47] Pollock A, St George B, Fenton M, Firkins L (2014). Top 10 research priorities relating to life after stroke--consensus from stroke survivors, caregivers, and health professionals. Int J Stroke.

